# Stretching Wormlike Chains in Narrow Tubes of Arbitrary Cross-Sections

**DOI:** 10.3390/polym11122050

**Published:** 2019-12-10

**Authors:** Ming Li, Jizeng Wang

**Affiliations:** Key Laboratory of Mechanics on Disaster and Environment in Western China, Ministry of Education, College of Civil Engineering and Mechanics, Lanzhou University, Lanzhou 730000, China; lim2015@lzu.edu.cn

**Keywords:** wormlike chain model, tube confinement, arbitrary cross-section, force-extension relation, stretching, GBR model, Brownian dynamics simulation

## Abstract

We considered the stretching of semiflexible polymer chains confined in narrow tubes with arbitrary cross-sections. Based on the wormlike chain model and technique of normal mode decomposition in statistical physics, we derived a compact analytical expression on the force-confinement-extension relation of the chains. This single formula was generalized to be valid for tube confinements with arbitrary cross-sections. In addition, we extended the generalized bead-rod model for Brownian dynamics simulations of confined polymer chains subjected to force stretching, so that the confinement effects to the chains applied by the tubes with arbitrary cross-sections can be quantitatively taken into account through numerical simulations. Extensive simulation examples on the wormlike chains confined in tubes of various shapes quantitatively justified the theoretically derived generalized formula on the force-confinement-extension relation of the chains.

## 1. Introduction

The statistical behaviors of semiflexible polymers confined in nano- and micro-tubes are fundamental problems in polymer physics and have been investigated both experimentally and theoretically for decades [1,2,3,4,5,6,7,8,9,10,11,12,13,14]. A thorough understanding of these problems is very important in the development of various application techniques that exploit the effects of confinement and stretching on polymers, including genome mapping [15,16], DNA sorting [17], and DNA denaturation mapping [18,19], etc.

The conformational behavior of a polymer in a tube is determined by the competition of three interactions: bending, excluded volume interacting, and confining [1]. Several distinct conformational regimes have been identified, each with its scaling properties for both configuration and free energy. For sufficiently narrow tubes, the polymer is highly extended and lies in the Odijk regime [20,21,22]. At the opposite extreme of large tubes, the polymer falls into the classic de Gennes regime [23]. Between these limits, rich physical regimes have been gradually revealed, which include the extended de Gennes [24,25,26] and the backfolded Odijk regimes, in which the latter was first predicted by Odijk [27,28] and later characterized by Muralidhar et al. [29,30] using techniques of computer simulations.

Although the effects of geometric constraint on the semiflexible chains are relatively well described, chain statistics under the simultaneous implementation of tube-like geometric confinement and force stretching has been much less understood [31]. However, there are still a few exceptions, for example, quantitative and compact formulae on the force-extension relation of a wormlike chain (WLC) confined in cylindrical and rectangular tubes [32,33,34,35] have been derived and numerically verified by using the Brownian dynamics simulations in terms of the Generalized-Bead-Rod model (GBR) [36]. Recently, these results have been successfully applied to characterize the experimental measurements on the thermal fluctuation of internal segments of DNA confined in a nanochannel [37], the theoretical description on entropically driven motion of polymers in nonuniform nanochannels [38], and the quantitative influence on how domain topology, stability, and translation speed may determine mechanical force generation on the ribosome [39], and so on. In addition, for slit-like confinements, Haan et al. [40] have derived an interpolation formula on the force-extension relation of DNA chains by introducing an effective dimensionality, which is considered as a generalization of the Marko-Siggia force-extension relation for WLCs [41] or a more general one given by Rosa et al. [42] valid for not only WLCs, but also freely jointed chains (FJCs); Also, Taloni et al. furnished a scaling framework to identify three distinct regimes (linear, nonlinear, bulk-like) attained by a slit-confined polymer subjected to an external pulling force [43].

Nevertheless, all these studies were aimed to understand the statics and dynamics of polymers in nanoslits and nanotubes with regular square or circular cross sections. It is still not clear how the more complex shapes of a confining tube may quantitatively influence the statistics of the confined polymer chains when a stretching force is simultaneously applied. This lack of knowledge contrasts with the increasing use of nanochannels with complex shapes in nanotechnologies. Most promisingly, theoretical research works have emerged, and techniques based on tubes with triangle cross-sections have been applied as polymeric devices for biomanipulation [44,45,46,47,48,49]. For example, Reinhart et al. [44] investigated the extension of DNA chains in isosceles triangular nanochannels by using Monte Carlo simulations of a touching-bead model. They found that the channel corners may enhance the extension, relative to a circular nanochannel of the same effective size. In addition, Manneschi et al. [45] also studied the conformations of DNA chains in the triangular, rectangular, and square channels by performing coarse-grained Monte Carlo simulations. Meanwhile, recent progresses in experimental technology have been able to fabricate nanofluidic channel systems with various cross-sections [47].

However, despite these progresses, it is still very urgent and necessary to develop a generalized theoretical model to quantitatively describe the statistical behavior of a polymer chain confined in channels with arbitrary cross-sections and simultaneously subjected to external stretching forces. In the present contribution, we theoretically investigated the conformations of WLCs confined in narrow tubes with arbitrary cross-sections and established a unique force-confinement-extension relation which is quantitatively applicable to nanotubes with any cross-sections. In order to validate the derived model, based on the semi-analytical method in the treatment of particle-wall interaction developed by Peters and Barenbrug [50], we extended our GBR model [36] to realize the Brownian dynamics simulations of WLCs confined in channels with arbitrary shapes.

## 2. Materials and Methods

### 2.1. Model

**Figure 1:**
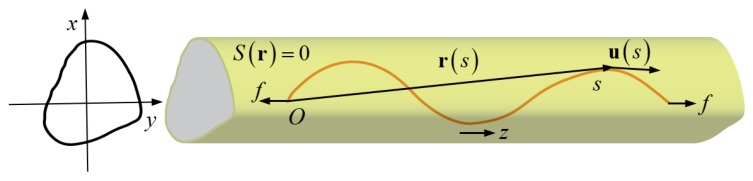
Schematic of a wormlike chain (WLC) confined in a tube with complex shape and subjected to force stretching.

Figure 1 shows the schematic of a WLC confined in a tube with an arbitrary convex cross-section and subjected to stretching by a tensile force. We assume that the geometric center of each cross-section of the tube forms the tube axis along a straight line. A set of Cartesian coordinates (x,y,z) are introduced so that the z axis is along the axis of the tube, and the profile of the cross-section of the tube at position z can be described by the equation, S(r)=0, where r=(x,y,z). Then Ω={r|S(r)<0} defines the set of all internal points enclosed by each cross-section boundary. For instance, the cross-section of a cylindrical tube of radius $l_{c}$ can be described by the equation $S\left(r\right)=\left[\left(x^{2}+y^{2}\right)/l_{c}^{2}\right]-1=0$, and Ω={r|S(r)<0} represents all the spatial points inside this cylindrical tube.

In addition, we assume that the confined chain is stretched by an applied tensile force, f=f k where k is the unit vector along the z-axis. The position vector at the arc length, s, of the chain can be expressed as

(1) $$r\left(s\right)=r_{z}\left(s\right)+z\left(s\right)k$$

where vector $r_{z}=\left[x\left(z\right),y\left(z\right)\right]$ is perpendicular to the z-axis at position z, and r∈Ω. The derivatives

(2) $$u=\frac{\partial r}{\partial s},u_{⊥}=\frac{\partial r_{z}}{\partial s},u_{∥}=\frac{dz}{ds}k$$

define the tangential vector and its components. As shown in [32,51], in the case of strong confinement, undulation of the chain due to thermal fluctuation is small so that $\left\|u_{⊥}\right\|\ll 1$. The inextensibility condition of the WLCs, ‖u‖=‖∂r/∂s‖=1 [52,53,54], together with Equation (2) leads to

(3) $$\frac{dz}{ds}=\left\|u_{∥}\right\|=1-\frac{1}{2}u_{⊥}^{2}+O\left(u_{⊥}^{4}\right)$$

Based on Equation (3), one can obtain the extension of the chain along the tube axis [51]

(4) $$R_{z}\equiv z(L)-z(0)\approx L-\frac{1}{2}\int_{0}^{L}u_{⊥}^{2}ds$$

We can further derive from Equations (1)–(3) that [32]

(5) $$\frac{\partial^{2}r}{\partial s^{2}}=\frac{\partial^{2}r_{z}}{\partial s^{2}}+\frac{d^{2}z}{\partial s^{2}}k=\frac{\partial }{\partial s}\left[u_{⊥}+O\left(u_{⊥}^{2}\right)k\right]\approx \frac{\partial u_{⊥}}{\partial s}$$

According to references [20,22,41], the Hamiltonian of the confined WLC without stretching force can be described as the summation of bending and potential energy as

(6) $$H=\frac{1}{2}L_{p}k_{B}T\int_{0}^{L}{\left(\frac{\partial^{2}r}{\partial^{2}s}\right)}^{2}ds+\int_{0}^{L}V\left(r\right)ds$$

in which

(7) $$V\left(r\right)=\{\begin{array}{cc} 0 & r\in \Omega \\ +\infty & \mathrm{otherwise} \end{array}$$

is the confinement potential per unit length due to the tube wall, and $L_{p}$ is the persistence length of the WLC. However, it can be very difficult to obtain analytical solutions of Equation (6) when hard wall boundary, Equation (7), is considered [55,56]. Instead, Burkhardt [55,56], Wang and Gao [32], and Wang and Li [35] found that the hard wall potential for confinements of cylindrical and rectangular tubes can be well approximated by effective harmonic potentials.

Following these studies, for the confinements of tubes with arbitrary cross-sections, we try to approximate the effect of hard wall boundaries by the harmonic potential

(8) $$V\left(r\right)=\frac{1}{2}\Xi r_{z}^{2}$$

where we assume that the parameter, Ξ, only depends on the shape/size of the confinement and the chain’s persistence length. In order to determine the parameter, Ξ, by considering Equations (2) and (5)–(8), we can have [32]

(9) $$H\approx \frac{1}{2}L_{p}k_{B}T\int_{0}^{L}{\left(\frac{\partial u_{⊥}}{\partial s}\right)}^{2}ds+\frac{\Xi }{2}\int_{0}^{L}{\left[\int_{0}^{s}u_{⊥}\left(\xi \right)d\xi \right]}^{2}ds$$

Following earlier investigations [32,41], we define the Fourier transform of $u_{⊥}$

(10) $${\tilde{u}}_{⊥}\left(\omega \right)=\int_{-\infty }^{\infty }e^{-i\omega s}u_{⊥}\left(s\right)ds$$

Then we have

(11) $$\int_{-\infty }^{\infty }e^{-i\omega s}\frac{\partial u_{⊥}}{\partial s}ds=i\omega {\tilde{u}}_{⊥},\;\;\;\;\;\;\;\;\;\int_{-\infty }^{\infty }e^{-i\omega s}\left[\int_{0}^{s}u_{⊥}\left(\xi \right)d\xi \right]ds=\frac{i}{\omega }{\tilde{u}}_{⊥}$$

Based on the Parseval theorem in mathematics, Equation (9) can be equivalently rewritten as

(12) $$\frac{H}{k_{B}T}=\frac{1}{4\pi }\int_{-\infty }^{\infty }\left(L_{p}\omega^{2}+\frac{\Xi }{k_{B}T}\frac{1}{\omega^{2}}\right){\tilde{u}}_{⊥}^{2}d\omega$$

The average energy contributed by each mode [32,41] is written as

(13) $$\frac{\langle H_{\omega }\rangle }{k_{B}T}=\frac{1}{2}\left(L_{p}\omega^{2}+\frac{\Xi }{k_{B}T}\frac{1}{\omega^{2}}\right)\langle {\tilde{u}}_{⊥}^{2}\rangle$$

According to the Equipartition theorem in statistical physics [41,57], $\langle H_{\omega }\rangle$ is equal to $k_{B}T$ for two degrees of freedom, then Equation (13) becomes

(14) $$\langle {\tilde{u}}_{⊥}^{2}\rangle =\frac{2}{L_{p}\omega^{2}+\Xi /\left(k_{B}T\omega^{2}\right)}$$

Using the Parseval theorem once again, we have

(15) $$\frac{1}{L}\int_{0}^{L}\langle u_{⊥}^{2}\rangle ds=\frac{1}{2\pi }\int_{-\infty }^{\infty }\langle {\tilde{u}}_{⊥}^{2}\rangle d\omega =2^{-1/2}{\left(\frac{\Xi L_{p}^{3}}{k_{B}T}\right)}^{-1/4}$$

According to Equations (4) and (15), we can obtain the average extension of the confined chain $R_{∥0}=\langle R_{z}\rangle$ without stretching force as

(16) $$\frac{R_{∥0}}{L}=1-2^{-3/2}{\left(\frac{\Xi L_{p}^{3}}{k_{B}T}\right)}^{-1/4}$$

Eventually, we can determine Ξ from Equation (16) as

(17) $$\Xi =2^{-6}{\left(1-\frac{R_{∥0}}{L}\right)}^{-4}\frac{k_{B}T}{L_{p}^{3}}$$

Equation (17) shows the relation between the “spring” constant of the effective harmonic potential, Ξ, and the extension of the chain due to confinement without stretching force.

We further assume that Equation (17) is still valid when a stretching force, f=fk, is applied to the confined chain. In this case, the Hamiltonian becomes [20,41]

(18) $$H=\frac{1}{2}L_{p}k_{B}T\int_{0}^{L}{\left(\frac{\partial^{2}r}{\partial^{2}s}\right)}^{2}ds-f\cdot \left[r\left(L\right)-r\left(0\right)\right]+\int_{0}^{L}V\left(r_{⊥}\right)ds$$

By noting that

(19) $$f\cdot \left[r\left(L\right)-r\left(0\right)\right]=f\left[z\left(L\right)-z\left(0\right)\right]\approx fL-\frac{f}{2}\int_{0}^{L}u_{⊥}^{2}ds$$

and discarding the constant term, Equation (18) can be approximately expressed as [32]

(20) $$H\approx \frac{1}{2}L_{p}k_{B}T\int_{0}^{L}{\left(\frac{\partial u_{⊥}}{\partial s}\right)}^{2}ds+\frac{f}{2}\int_{0}^{L}u_{⊥}^{2}ds+\frac{\Xi }{2}\int_{0}^{L}{\left[\int_{0}^{s}u_{⊥}\left(\xi \right)d\xi \right]}^{2}ds$$

Similarly, we can obtain [32],

(21) $$\frac{1}{L}\int_{0}^{L}\langle u_{⊥}^{2}\rangle ds=\frac{1}{\sqrt{fL_{p}/k_{B}T+2\sqrt{\Xi L_{p}^{3}/k_{B}T}}}$$

Further, we have [32]

(22) $$R_{∥}=\langle z\left(L\right)-z\left(0\right)\rangle \approx L-\frac{L}{2}\frac{1}{\sqrt{fL_{p}/k_{B}T+2\sqrt{\Xi L_{p}^{3}/k_{B}T}}}$$

Insertion of Equation (17) into Equation (22) yields

(23) $$\frac{R_{∥}}{L}\approx 1-\frac{1}{\sqrt{4fL_{p}/k_{B}T+{\left(1-R_{∥0}/L\right)}^{-2}}}$$

Equation (23) shows the force-confinement-extension relation for the WLC confined in a tube with arbitrary cross-section and simultaneously subjected to force stretching. From Equation (23), we can see that, as $fL_{p}/k_{B}T\to 0$, we have $R_{∥}/L\to R_{∥0}/L$, and as $fL_{p}/k_{B}T\to \infty$, Equation (23) can be reduced to

(24) $$\frac{R_{∥}}{L}\to 1-\frac{1}{2\sqrt{fL_{p}/k_{B}T}}$$

which is exactly the classic force-extension relation for large forces. This fact implies that Equation (23) is exact when the tensile force is either very small or very large. For these two extremes, the normalized tube size only influences how small or large the tensile force in which Equation (23) can give an accurate prediction.

On the other hand, as the tube size is infinitely small, we have $R_{∥0}/L\to 1$. Then the tensile forces become irrelevant to the extension of the confined chains due to the inextensibility of the WLCs. As $R_{∥0}/L\to 0$, Taylor expansion of Equation (23) in terms of $R_{∥0}/L$ yields by omitting higher order terms.

(25) $$\frac{R_{∥}}{L}\approx 1-\frac{1}{\sqrt{4fL_{p}/k_{B}T+1}}+\frac{R_{∥0}/L}{\sqrt{{\left(4fL_{p}/k_{B}T+1\right)}^{3}}}$$

### 2.2. Brownian Dynamics Simulations

In order to verify the derived force-confinement-extension relation, we consider the GBR model established by Wang and Gao [36] for the Brownian dynamics simulations of confined WLCs under stretching. This model has been successfully applied to the quantitative analysis of statistical properties of polymers confined on spherical surfaces [58] and in cylindrical and rectangular tubes [32,33,35]. In the GBR model, a WLC is depicted as N identical virtual beads of radius, a, connected by N−1 inextensible rods of length b with the unit tangent vectors, $u_{j}$ ($\left|u_{j}\right|=1,j=1,2,\ldots ,N-1$). The contour length of the polymer chain is L=(N−1)b. The N virtual beads with coordinates, $r_{j}=\left(x_{j},y_{j},z_{j}\right)$ (j=1,2,…,N), are introduced for simulating hydrodynamic interactions between different sections.

Motion equation of the N beads can be expressed as follows in terms of the second Newton law

(26) $$M\frac{d^{2}\hat{r}}{dt^{2}}+C\frac{d\hat{r}}{dt}+F=\zeta$$

where M is a 3N×3N diagonal matrix containing the mass of each bead, $\hat{r}$ is the 3N position vector at time t, C is the configuration-dependent friction vector, F is the configuration-dependent collective vector of internal and external forces including bending, stretching, and geometrical constraining [59,60,61], and ζ is the randomly fluctuating force exerted on the beads by the surrounding fluid.

If the inertia effect is negligible, we have the overdamped equation of motion in the form

(27) $$C\frac{d\hat{r}}{dt}+F=\zeta$$

Equation (27) can be readily solved by using the Euler method

(28) r^(n+1)=r^(n)+ΔtkBTD(n)F(n)+ξ(n)

where Δt is the time step, subscript “(*n*)” represents the *n*th time step, $D=k_{B}TC^{-1}$ is the translational diffusion matrix (see Ref. [36] for the detail), $\xi_{\left(n\right)}$ represents the collective random displacements at the *n*th time step generated from a Gaussian distribution with zero mean and variance

(29) 〈ξξ(n)(n′)〉=2D(n)Δtδnn′

where $\delta_{nn^{\prime }}$ is the Kronecker delta symbol. As a simulation model of discrete WLCs, a linear constraint solver is applied to realize the rod length constraint [61]

(30) $$g_{i}(r)=\left\|r_{i+1}-r_{i}\right\|-b=0,i=1,2,\ldots ,N-1$$

If we define $B=\left\{\partial g_{i}\left(\hat{r}\right)/\partial \hat{r},i=1,2,\ldots ,N-1\right\}$, and add the constraints to the motion equation through the Lagrange multiplier vector, eventually we obtain the following equation to determine the new position vector ${\hat{r}}_{\left(n+1\right)}$ of the N beads

(31) $${\hat{r}}_{\left(n+1\right)}=\Lambda \left({\hat{r}}_{\left(n\right)}+\frac{\Delta t}{k_{B}T}D_{\left(n\right)}F_{\left(n\right)}+\xi_{\left(n\right)}\right)+T_{\left(n\right)}d$$

where $d={\left\{b,b,\ldots ,b\right\}}^{T}$, $T_{\left(n\right)}=D_{\left(n\right)}B_{\left(n\right)}^{T}{\left(B_{\left(n\right)}^{}D_{\left(n\right)}B_{\left(n\right)}^{T}\right)}^{-1}$, and $\Lambda =I-T_{\left(n\right)}B_{\left(n\right)}$ as a projection matrix which sets the constraints.

In the GBR simulation model proposed by Wang and Gao [36], a semi-analytical method on the random motion of a bead near a reflecting wall established by Peters and Barenrug [50] was taken into account for the cylindrical tube confinements. In this study, as tubes with arbitrary cross-sections are considered, therefore extension to the original GBR model is necessary to realize the complex spatial confinement. To this end, we assume that profile of the tube can be expressed as

(32) S(r)=0

where r=(x,y,z) represents the position vector of a point on the tube surface. Considering the *j*th bead in the chain with current position, $r_{\left(n\right)j}=\left(x_{\left(n\right)j},y_{\left(n\right)j},z_{\left(n\right)j}\right)$, the distance from this bead to the point (x,y,z) on the tube surface can be given by

(33) $$d_{\left(n\right)j}(r)=\left\|r-r_{\left(n\right)j}\right\|$$

In the following, we derive the minimum value of $d_{\left(n\right)j}$ as a function of position vector, r. To do this, we introduce the Lagrange function [62] as follows

(34) $$ℒ\left(r,\lambda \right)=d_{\left(n\right)j}(r)+\lambda S\left(r\right)$$

where λ is the Lagrange multiplier. The gradient of the function, ℒ(r,λ), can be given by

(35) $$\nabla_{r,\lambda }ℒ=\left(\frac{\partial ℒ}{\partial r},\frac{\partial ℒ}{\partial \lambda }\right)=\left(\frac{\partial d_{\left(n\right)j}}{\partial r}+\lambda \frac{\partial S}{\partial r},S\right)$$

Setting $\nabla_{r,\lambda }ℒ=$, so that

(36) $$\frac{\partial }{\partial r}\left\|r-r_{\left(n\right)j}\right\|+\lambda \frac{\partial S}{\partial r}=0,S(r)=0$$

Solution of Equation (36) can give the position on the tube surface, $r=r_{s}=\left(x_{s},y_{s},z_{s}\right)$, so that $d_{\left(n\right)j}(r_{s})$ reach its minimum value. In the case that S(r) is piecewise continuous, we simply neglect the effect of undifferentiable connecting lines on the surface.

For a one-dimensional bead moving in its coordinate, X, Peters and Barenrug [50] studied the effect of a flat reflecting wall at position, X=0, based on the solution of the corresponding Fokker–Planck equation for the probability distribution of the bead position. They derived the stochastic movement of the bead after one time-step as

(37) $$\Delta X=f_{1}\left(\frac{X}{\sqrt{D\Delta t}}\right)\sqrt{D\Delta t}+f_{2}\left(\frac{X}{\sqrt{D\Delta t}}\right)\sqrt{D}\Delta \beta$$

where D represents the diffusion coefficient of the bead, and Δβ the random variable with first moment 0 and second moment Δt. The functions $f_{1}\left(.\right)$ and $f_{2}\left(.\right)$ have the forms of [50]

(38) $$f_{1}\left(X\right)=\frac{2}{\sqrt{\pi }}\exp\left(-\frac{X^{2}}{4}\right)-X\left[1-\mathrm{erf}\left(\frac{X}{2}\right)\right]$$

(39) $$f_{2}\left(X\right)=\sqrt{2+X^{2}-\left[X\mathrm{erf}\left(\frac{X}{2}\right)+\frac{2}{\sqrt{\pi }}\exp\left(-\frac{X^{2}}{4}\right)\right]}$$

We can imagine that as long as the time step is sufficiently small, the local curvature of the tube surface will be no longer significant to the reflecting effect of the tube wall. Therefore, similar to the case of the flat reflecting wall, when the bead is sufficiently close to the tube wall, say $\left\|r_{\left(n\right)j}-r_{s}\right\|<\sqrt{5D\Delta t}$, we may set the next stochastic movement of the *j*th bead due to the reflecting tube surface, S(r), as [36,50]

(40) $$\Delta r_{\left(n\right)}^{\mathrm{wall}}=\{\begin{array}{cc} \frac{r_{\left(n\right)j}-r_{s}}{\left\|r_{\left(n\right)j}-r_{s}\right\|}\left[f_{1}\left(\frac{\left\|r_{\left(n\right)j}-r_{s}\right\|}{\sqrt{D\Delta t}}\right)\sqrt{D\Delta t}+f_{2}\left(\frac{\left\|r_{\left(n\right)j}-r_{s}\right\|}{\sqrt{D\Delta t}}\right)\sqrt{D}\Delta \beta \right], & \;\left\|r_{\left(n\right)j}-r_{s}\right\|<\sqrt{5D\Delta t}\\ 0, & \;\left\|r_{\left(n\right)j}-r_{s}\right\|\geq \sqrt{5D\Delta t} \end{array}$$

Taking into account the confinement effect in terms of Equation (40), then the positions of *N* beads at the next time step can be determined by

(41) $${\hat{r}}_{\left(n+1\right)}=\left(I-T_{\left(n\right)}B_{\left(n\right)}\right)\left({\hat{r}}_{\left(n\right)}+\Delta {\hat{r}}_{\left(n\right)}^{\mathrm{wall}}+\frac{\Delta t}{k_{B}T}D_{\left(n\right)}F_{\left(n\right)}+\xi_{\left(n\right)}\right)+T_{\left(n\right)}d$$

In the following, we show several examples of tubes with different profiles.

(a) Polygonal tubes

For a straight tube with polygon cross-sections, we assume that the polygon is *n*-sided and locates in the x−y plane at the position, z, coordinate of its *j*th vertex is denoted as $\left(x_{j},y_{j},z\right)$, j=1,2,…,n. Then, the whole profile of such a tube can be expressed as

(42) $$S(r)=y-\left(x-x_{m}\right)\frac{y_{m+1}-y_{m}}{x_{m+1}-x_{m}}-y_{m}=0,x\in \left(x_{m},x_{m+1}\right),m=1,2,\ldots ,n$$

where $x_{n+1}=x_{1},\;\;\;\;y_{n+1}=y_{1}$.

(b) Elliptic tubes

For straight tubes with elliptic cross-sections, their profiles can be described as

(43) $$S\left(r\right)=\frac{x^{2}}{R_{1}^{2}}+\frac{y^{2}}{R_{2}^{2}}-1=0$$

where $R_{1}$ and $R_{2}$ are positive real constants.

(c) Wavy tubes

For periodic wavy tubes with effective wave length l, assuming that profile of the first wavelength can be defined by $S_{0}(r)=0$, then we can express its overall profile as

(44) $$S\left(r\right)=S_{0}(x,y,z-nl)=0,n\in Z$$

## 3. Results and Discussion

Based on the extended GBR model [32,36], Brownian dynamics simulations were performed for WLCs confined in tubes of different shapes. In all the simulations, the chains were initially set in straight configurations. Tube confinements and stretching forces were then applied during the chains’ relaxation. We recorded the average extension of the chain along the z-axis, R=z(0,t)−z(L,t), at a fixed time increment. For each simulation, the average extension of the corresponding WLC was obtained by first averaging over time and then over numerous independent trajectories obtained by using different random seeds. In all the simulations, we set the persistence length of the WLCs as *L*_p_ = 50 nm, the viscosity of the solvent, $\eta =8.904\times 10^{-4}\;\mathrm{Pa}\cdot s$, the absolute temperature, T=298 K, bead radius, 0.98 nm, bond length, 2.0 nm, time step, 6.0 ps, contour length, 100–120 nm and total simulation time, 60–200 μs. For each simulation, ensemble average was performed over 14–200 different trajectories with different random seeds. As we have shown in Appendix A, the extended GBR model can realize the spatial confinement of tubes with complex shapes and the equilibrium states of confined and stretched WLCs can be reached within a few tens microseconds.

**Figure 2:**
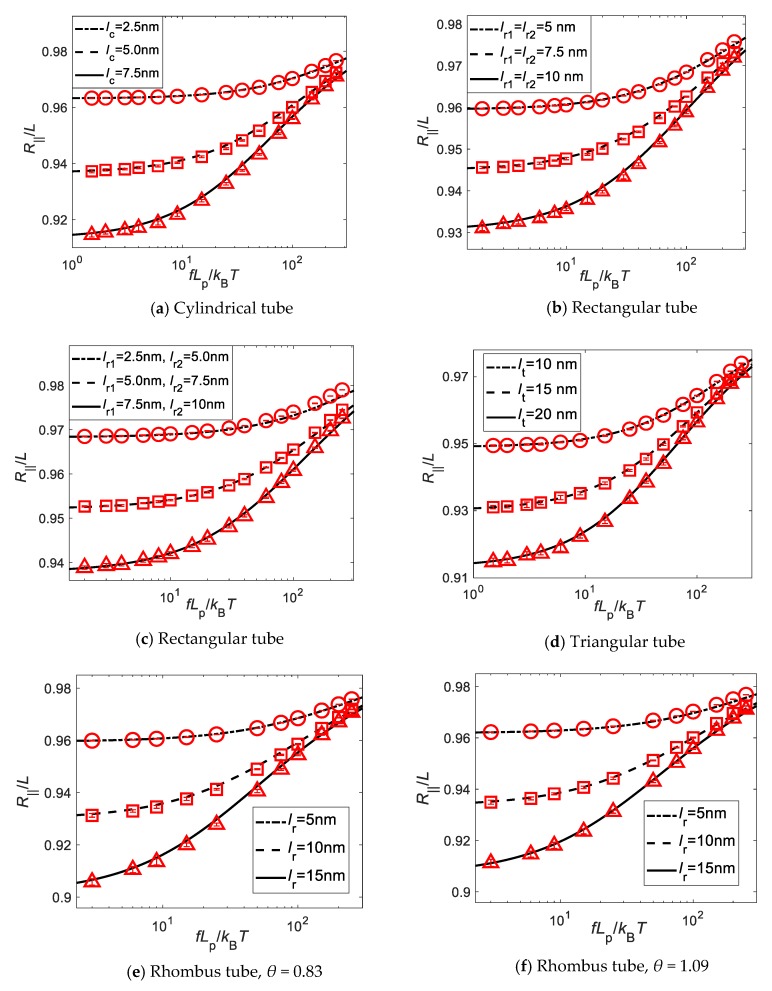

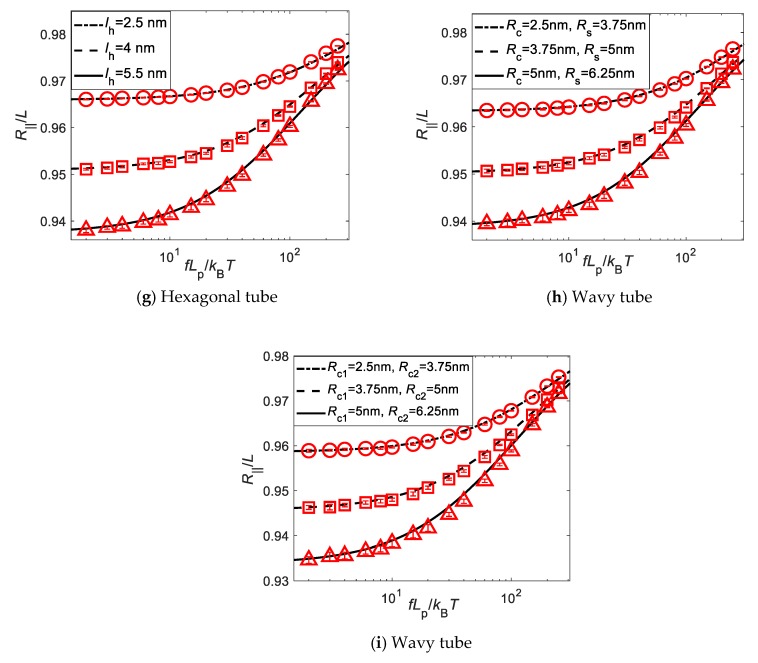
Comparison of simulation results on the force-confinement-extension relations of confined WLCs and their corresponding theoretical predictions based on Equation (23) under confinements of tubes with various cross-sections.

To numerically verify the derived force-confinement-extension relation in Equation (23) for the confined and stretched WLCs, in the following, we consider the Brownian dynamics simulations of the chains confined in tubes of various shapes. The simulation results are represented by hollow circles, squares, rhombuses, and triangles, and corresponding theoretical predictions based on Equations (23) and (25) by solid, dashed and dash-dotted lines. Figure 2 illustrates the relation between the applied normalized stretching force, $fL_{p}/k_{B}T$, and the normalized extension of the confined WLCs, where relevant geometrical and simulation parameters are listed in Table 1. From Figure 2, we can see that theoretical predictions based on Equation (23) are in good agreement with all the simulation results for a large range of tensile forces.

**Figure 3:**
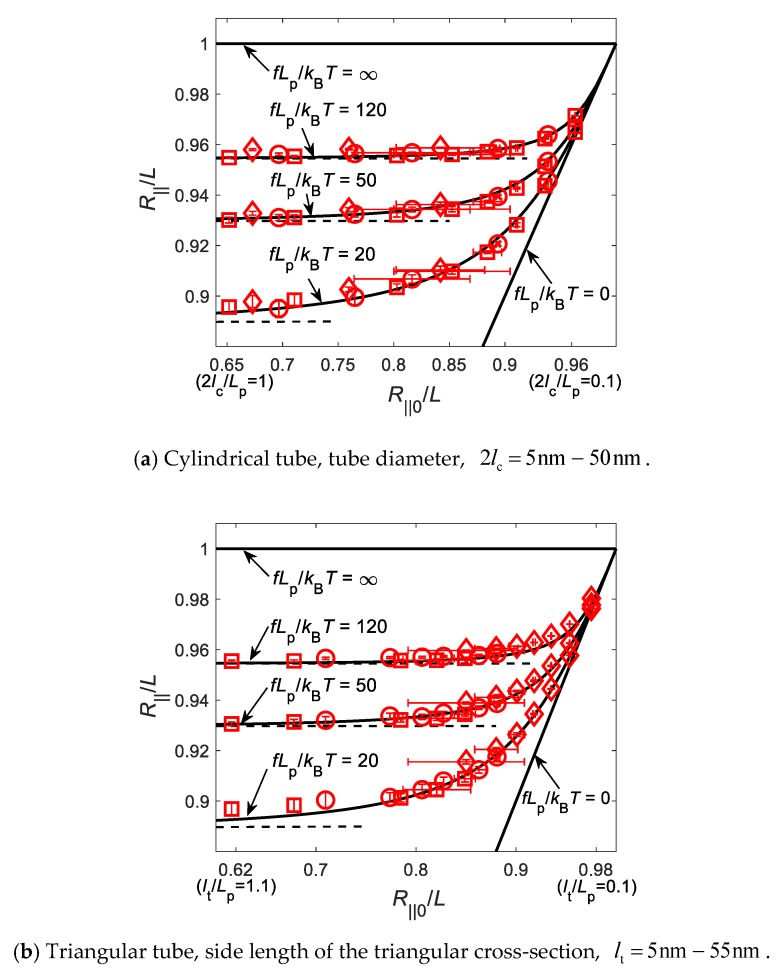
Comparison of simulation results on the force-confinement-extension relations of the confined WLCs and their corresponding theoretical predictions based on Equations (23) and (25) under confinements of tubes with cross-sections of (**a**) circular, and (**b**) triangular shapes. Note that the simulation data for $R_{∥0}/L<0.85$ show very large horizontal error bars, which are not plotted in the figure for clarity.

In order to investigate how the tube size or the unstretched extension, $R_{∥0}/L$, may influence the force-confinement-extension relation, we considered the confinements of tubes with circular and triangular cross-sections. We performed extensive Brownian dynamics simulations based on the GBR model to investigate the extension of the chains confined in tubes of various sizes and stretched by different tensile forces. Figure 3 shows the relation between the normalized extension, $R_{∥}/L$, under tensile force, $fL_{p}/k_{B}T$, and the unstretched extension, $R_{∥0}/L$, that decreases monotonically with the increase of tube size. Solid and dashed lines in Figure 3 are theoretical predictions based on Equations (23) and (25) under different tensile forces. In Figure 3a, hollow squares, circles and rhombuses represent the simulation results with simulation parameters of contour lengths, *L* = 100 nm, 150 nm, and 200 nm, total simulation times, 90 µs, 90 µs, and 150 µs, and total simulation trajectories, 69, 112, 69, respectively. In Figure 3b, the simulation results are still represented by the hollow squares, circles and rhombuses, for the contour lengths, *L* = 100 nm, 150 nm, and 200 nm, total simulation times, 90 µs, 90 µs, and 300 µs, and total simulation trajectories, 84, 50, and 140, respectively. We can observe from Figure 3 that numerical simulations agree very well with the theoretical predictions for wide ranges of tensile forces and tube sizes, though the derivation of Equation (23) assumes strong confinement.

## 4. Conclusions

We studied the statistical mechanics behavior of WLCs confined in narrow tubes with arbitrary cross-sections and derived a generally applicable compact expression on the force-extension relation of the confined chains. We also extended our generalized bead-rod (GBR) model for Brownian dynamics simulations of confined polymer chains subjected to force stretching, so that the confinement effects arising by tubes with complex shapes to the chains can be quantitatively accounted for during the numerical simulations. Sufficiently large numbers of simulations on the WLCs confined in tubes of various shapes were performed, which quantitatively justified the theoretically derived generalized force-confinement-extension relation.

## Figures and Tables

**Table 1:** Tube geometries and simulation parameters.

Tube Geometries		Simulation Parameters
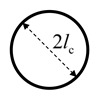	$S\left(r\right)=\frac{x^{2}+y^{2}}{l_{c}^{2}}-1$	Figure 2a $\begin{array}{l} l_{c}=2.5\;\mathrm{nm},5.0\;\mathrm{nm},7.5\;\mathrm{nm}\\ L=100\;\mathrm{nm},110\;\mathrm{nm},120\;\mathrm{nm}\\ \mathrm{Simulation}\mathrm{time}=90\;\mu s \end{array}$
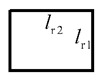	$\begin{array}{l} S(r)=y-\left(x-x_{m}\right)\frac{y_{m+1}-y_{m}}{x_{m+1}-x_{m}}-y_{m},\\ x_{m}<x<x_{m+1},m=1,2,3,4.\\ \left(x_{1},y_{1}\right)=-\left(x_{3},y_{3}\right)=\left(-l_{r1}/2,-l_{r2}/2\right),\\ \left(x_{2},y_{2}\right)=-\left(x_{4},y_{4}\right)=\left(l_{r1}/2,-l_{r2}/2\right). \end{array}$	Figure 2b $\begin{array}{l} l_{r1}=l_{r2}=5\;\mathrm{nm},7.5\;\mathrm{nm},10\;\mathrm{nm}\\ L=100\;\mathrm{nm},110\;\mathrm{nm},120\;\mathrm{nm}\\ \mathrm{Simulation}\mathrm{time}=90\;\mu s \end{array}$
		Figure 2c $\begin{array}{l} l_{r1}=2.5\;\mathrm{nm},5\;\mathrm{nm},7.5\;\mathrm{nm}\\ l_{r2}=5\;\mathrm{nm},7.5\;\mathrm{nm},10\;\mathrm{nm}\\ L=100\;\mathrm{nm},110\;\mathrm{nm},120\;\mathrm{nm}\\ \mathrm{Simulation}\mathrm{time}=90\;\mu s \end{array}$
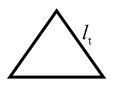	$\begin{array}{l} S(r)=y-\left(x-x_{m}\right)\frac{y_{m+1}-y_{m}}{x_{m+1}-x_{m}}-y_{m},\\ x_{m}<x<x_{m+1},m=1,2,3.\\ \left(x_{1},y_{1}\right)=\left(-l_{t}/2,-\sqrt{3}l_{t}/6\right),\\ \left(x_{2},y_{2}\right)=\left(l_{t}/2,-\sqrt{3}l_{t}/6\right),\\ \left(x_{3},y_{3}\right)=\left(0,\sqrt{3}l_{t}/3\right). \end{array}$	Figure 2d $\begin{array}{l} l_{t}=10\;\mathrm{nm},15\;\mathrm{nm},20\;\mathrm{nm}\\ L=100\;\mathrm{nm},110\;\mathrm{nm},120\;\mathrm{nm}\\ \mathrm{Simulation}\mathrm{time}=90\;\mu s \end{array}$
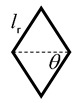	$\begin{array}{l} S(r)=y-\left(x-x_{m}\right)\frac{y_{m+1}-y_{m}}{x_{m+1}-x_{m}}-y_{m},\\ x_{m}<x<x_{m+1},m=1,2,3,4.\\ \left(x_{1},y_{1}\right)=-\left(x_{3},y_{3}\right)=\left(-l_{r}\sin\theta ,0\right),\\ \left(x_{2},y_{2}\right)=-\left(x_{4},y_{4}\right)=\left(0,-l_{r}\cos\theta \right). \end{array}$	Figure 2e $\begin{array}{l} \theta =0.83,L=100\mathrm{nm}\\ l_{r}=5\mathrm{nm},10\mathrm{nm},15\mathrm{nm}\\ \mathrm{Simulation}\mathrm{time}=120\;\mu s \end{array}$
		Figure 2f $\begin{array}{l} \theta =1.09,L=100\;\mathrm{nm}\\ l_{r}=5\;\mathrm{nm},10\;\mathrm{nm},15\;\mathrm{nm}\\ \mathrm{Simulation}\mathrm{time}=90\;\mu s \end{array}$
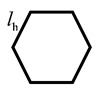	$\begin{array}{l} S(r)=y-\left(x-x_{m}\right)\frac{y_{m+1}-y_{m}}{x_{m+1}-x_{m}}-y_{m},\\ x_{m}<x<x_{m+1},m=1,2,3,4,5,6.\\ \left(x_{1},y_{1}\right)=-\left(x_{4},y_{4}\right)=\left(-l_{h},0\right),\\ \left(x_{2},y_{2}\right)=-\left(x_{5},y_{5}\right)=\left(-l_{h}/2,-\sqrt{3}l_{h}/2\right),\\ \left(x_{3},y_{3}\right)=-\left(x_{6},y_{6}\right)=\left(l_{h}/2,-\sqrt{3}l_{h}/2\right). \end{array}$	Figure 2g $\begin{array}{l} l_{h}=2.5\mathrm{nm},4\mathrm{nm},5.5\mathrm{nm}\\ L=100\;\mathrm{nm},110\;\mathrm{nm},120\;\mathrm{nm}\\ \mathrm{Simulation}\mathrm{time}=60\;\mu s \end{array}$
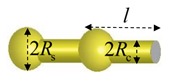	$\begin{array}{l} S\left(r\right)=S_{0}(x,y,z-nl),n\in Z\\ S_{0}\left(r\right)=\left\{ \begin{array}{l} x^{2}+y^{2}+z^{2}-R_{s}^{2},z\in \left(-d,d\right)\\ x^{2}+y^{2}-R_{c}^{2},\;\;\;z\in \left(d,l-d\right) \end{array}\right.\\ \mathrm{where}d=\sqrt{R_{s}^{2}-R_{c}^{2}}\mathrm{and}R_{s}>R_{c}. \end{array}$	Figure 2h $\begin{array}{l} R_{c}=2.5\;\mathrm{nm},3.75\;\mathrm{nm},5\;\mathrm{nm}\\ R_{s}=3.75\;\mathrm{nm},5\mathrm{nm},6.25\;\mathrm{nm}\\ l=20.6\;\mathrm{nm},21.6\;\mathrm{nm},22.5\;\mathrm{nm}\\ L=100\;\mathrm{nm},110\;\mathrm{nm},120\;\mathrm{nm}\\ \mathrm{Simulation}\mathrm{time}=60\;\mu s \end{array}$
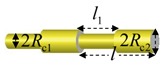	$\begin{array}{l} S\left(r\right)=S_{0}(x,y,z-nl),n\in Z\\ S_{0}\left(r\right)=\left\{ \begin{array}{l} x^{2}+y^{2}-R_{c1}^{2},z\in \left(0,l_{1}\right)\\ x^{2}+y^{2}-R_{c2}^{2},z\in \left(l_{1},l-l_{1}\right) \end{array}\right. \end{array}$	Figure 2i $\begin{array}{l} R_{c1}=2.5\;\mathrm{nm},3.75\;\mathrm{nm},5\;\mathrm{nm}\\ R_{c2}=3.75\;\mathrm{nm},5\;\mathrm{nm},6.25\;\mathrm{nm}\\ l_{1}=30\;\mathrm{nm},l=60\;\mathrm{nm},L=100\;\mathrm{nm}\\ \mathrm{Simulation}\mathrm{time}=60\;\mu s \end{array}$

**Figure A1:**
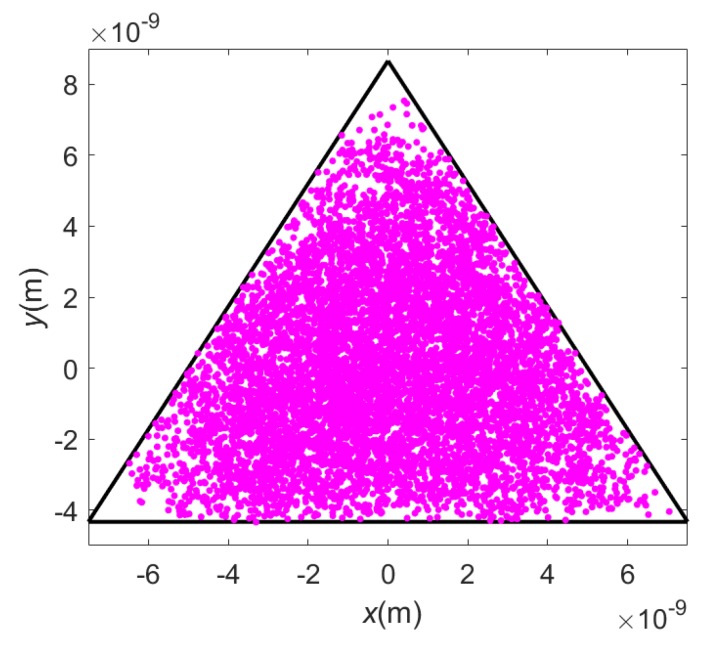
Recorded bead positions in the cross-section plane for a stretched WLC confined in a triangular tube during the Brownian dynamics simulations based on the extended generalized bead-rod (GBR) model. Triangle side length, 15 nm; Normalized stretching force, $25k_{B}T/L_{p}$.

**Figure A2:**
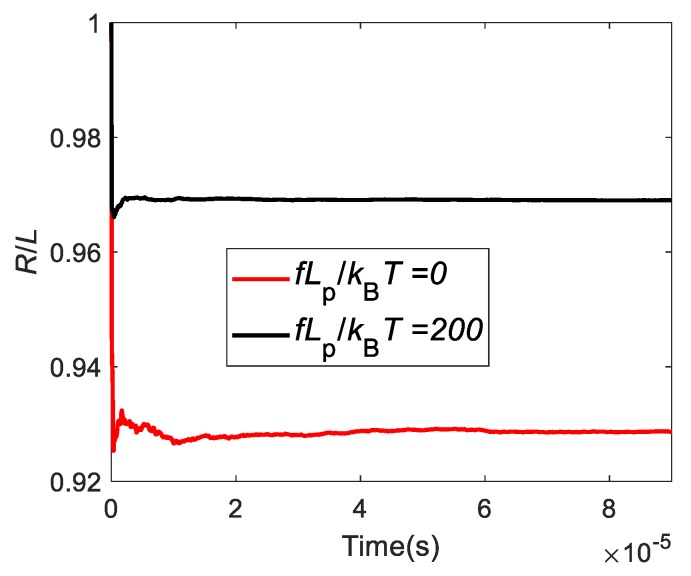
Evolution of ensemble average of the WLC extension, which is confined in a triangular tube with cross-section side length, 15 nm.

## Appendix A

In order to verify if the extended GBR model can realize the spatial confinement of tubes with complex shapes, we consider a WLC to be confined in the tube with cross-section of the regular triangle and subjected to a stretching force.

During the simulations, we record (x,y) coordinates of all the beads on the chain per every 0.6 μs. The total simulation time is set as 90 μs. Then we plot all the recorded positions of the beads in the x−y plane to see if all the positions are well confined in the region of the cross-section of the tube. Figure A1 shows the positions of all the beads on the x−y plane. It can be seen from Figure A1 that all the beads are well located inside the triangle domain, implying that the extended GBR model is successful in simulations of stretched chains in complex tube confinements.

In addition, Figure A2 shows the evolution of the ensemble average of the end-to-end distance of the chain under different stretching forces. Each curve in Figure A2 is averaged over 14 different trajectories with different random seeds. It can be seen from this figure that the equilibrium states can be reached within about 20 μs.
